# IGF and mTOR pathway expression and in vitro effects of linsitinib and mTOR inhibitors in adrenocortical cancer

**DOI:** 10.1007/s12020-019-01869-1

**Published:** 2019-03-05

**Authors:** Maria Cristina De Martino, Peter M. van Koetsveld, Richard A. Feelders, Wouter W. de Herder, Fadime Dogan, Joseph A. M. J. L. Janssen, Davine Hofste op Bruinink, Claudia Pivonello, A. Marlijn Waaijers, Annamaria Colao, Ronald R. de Krijger, Rosario Pivonello, Leo J. Hofland

**Affiliations:** 1 Department of Internal Medicine, Division Endocrinology, Erasmus Medical Center, Rotterdam, The Netherlands; 2 Dipartimento di Medicina Clinica e Chirurgia, Università Federico II, Naples, Italy; 3 Departments of Pathology, Erasmus Medical Center, Rotterdam, and University Medical Center Utrecht, Utrecht, The Netherlands

**Keywords:** Adrenal, Linsitinib, IGF, Insulin receptor, Adrenocortical cancer

## Abstract

**Purpose:**

The IGF and mTOR-pathways are considered as potential targets for therapy in patients with adrenocortical carcinoma (ACC). This study aims to describe the IGF pathway in ACC and to explore the response to the combined treatment with the IGF1R/IR inhibitor linsitinib, and mTOR inhibitors (sirolimus and everolimus) in in vitro models of ACC.

**Methods:**

The protein expression level of IGF2, IGF1R and IGF2R was evaluated by immunohistochemistry in 17 human ACCs and the mRNA expression level of *IGF1*, *IGF2*, *IGF1R*, *IR* isoforms *A* and *B*, *IGF2R*, *IGF-Binding-Proteins[IGFBP]-1, 2, 3* and *6* was evaluated by RT-qPCR in 12 samples. In H295R and HAC15 ACC cell lines the combined effects of linsitinib and sirolimus or everolimus on cell survival were evaluated.

**Results:**

A high protein expression of IGF2, IGF1R and IGF2R was observed in 82, 65 and 100% of samples, respectively. A high relative expression of *IGF2* mRNA was found in the majority of samples. The mRNA levels of the *IRA* were higher than that of *IRB* and *IGF1R* in the majority of samples (75%). Linsitinib inhibits cell growth in the H295R and HAC15 cell lines and, combined with sirolimus or everolimus, linsitinib showed a significant additive effect.

**Conclusions:**

In addition to IGF2 and IGF1R, ACC express IGF2R, IRA and several IGFBPs, suggesting that the interplay between the different components of the IGF pathway in ACC could be more complex than previously considered. The addition of mTOR inhibitors to linsitinib may have stronger antiproliferative effects than linsitinib alone.

## Introduction

Malignant tumors of the adrenal cortex, defined as adrenocortical carcinoma (ACC), are rare but aggressive cancers for which new treatment options are required [1–4]. Although most ACCs are sporadic, ACCs rarely develop in the context of certain genetic syndromes such as the Beckwith–Wiedemann syndrome (BWS), Li-Fraumeni and familial colorectal polyposis. The study of these syndromes has supported the potential role of some molecular pathways in ACC pathogenesis [5]. Particularly, the BWS is a genetic syndrome associated with childhood ACC, other childhood tumors and a somatic overgrowth syndrome in which deregulation of imprinted genes on chromosomal locus 11p15 leads to biallelic expression of *IGF2* [5, 6]. Although the estimated prevalence of BWS in patients with ACC is very low and restricted to the childhood [5, 7], IGF2 has been reported to be over-expressed in about 70–90% of sporadic ACCs as compared to normal adrenals or benign adrenocortical tumors [8–17]. Therefore, the insulin-like growth factor (IGF) system is considered as a promising target for new medical treatment options in ACC [11, 14, 18]. The IGF system participates in the regulation of growth, lifespan and metabolism and includes circulating ligands, exerting their effects as endocrine and/or paracrine factors [insulin, IGF1 and IGF2 (IGFs)]; binding proteins (IGFBP1-6 that modulate the bioavailability of IGFs) and multiple receptors [19]. Among the receptors, the IGF1 receptor (IGF1R) and the insulin receptor isoforms A and B (IRA and IRB) are tyrosine-kinase receptors. The mannose 6-phosphate/insulin-like growth factor 2 receptor (IGF2R) is a scavenger receptor involved in the internalization and degradation of IGF2. In adult humans, insulin predominantly exerts metabolic effects through the activation of IRB, whereas IGFs, particularly IGF1, mainly exerts growth-stimulating effects through the activation of IGF1R receptors. IRA is predominantly expressed during fetal development when it is an important mediator of pro-growth effects of insulin and IGFs. IRA and its expression in malignant tumor tissue has been suggested to be involved in cancer development [19, 20]. Currently, the efficacy of several IGF1R and IGF1R/IR inhibitors is evaluated in clinical trials, alone or in combination with other agents for the treatment of several malignant disorders [21–23]. mTOR is a protein kinase of the phosphatidylinositol 3-kinase (PI3K)/Akt/mTOR signaling pathway and plays a pivotal role in cell growth, metabolism and proliferation, by mediating the effects of various growth factors, including the IGFs [24]. The mTOR pathway is considered a target for antineoplastic therapy in several malignancies and it has recently been proposed as a target for ACC treatment [25–28].

This study aims at describing the IGF pathway in ACC and to explore the in vitro response to the combined treatment with a dual IGF1R/IR inhibitor (linsitinib) and the mTOR inhibitor (sirolimus) in an in vitro model of ACC using ACC cell lines.

## Materials and methods

### Subjects

Seventeen ACCs and 6 normal adrenal tissue samples (NA) samples were used for this study. Fresh tissue was snap frozen within 60 minutes after surgical removal. NA samples were collected for in vitro studies from adrenalectomy (NA) due to renal cell carcinoma. This study was approved by the Medical Ethics Committee of the Erasmus MC and all patients gave written informed consent.

The following clinical parameters were recorded in all patients: date of diagnosis, age, gender, ENSAT stage [29], Weiss score (assessed by an expert pathologist in adrenal disease [RRdK]) [30], mitotic count (as defined by the presence number of mitoses equal or higher than 5 in 50 high-power fields), hormonal status and type of hormonal secretion (cortisol and/or androgens and/or estrogens and/or mineralocorticoids) [31].

### Total RNA isolation and quantitative RT-PCR (RT-qPCR)

From snap frozen adrenal tissues (available for 12 ACCs cases and 6 NA cases), total RNA was isolated using a commercially available kit (High Pure RNA Tissue kit; Roche, Almere, The Netherlands).

Total RNA from the human ACC cell line NCI-H295R (H295R) was used as a positive control.

The cDNA synthesis from total RNA and quantitative PCR were performed as previously described [25]. mRNA expression of IGF1, IGF2, IGF1R, IRA, IRB, IGF2R, IGFBP 1, 2, 3 and 6 and of the housekeeping gene hypoxanthine phosphoribosyltransferase (HPRT) was evaluated by RT-qPCR in human ACC tissue samples, depending on the availability of frozen tissues.

The primers and probes were purchased from Sigma-Aldrich (Zwijndrecht, The Netherlands) and are reported in the Supplemental table 1. Samples were normalized to the expression of HPRT. PCR efficiencies (E) were calculated for the primer-probe combinations used [32]. The relative expression of genes was calculated using the comparative threshold method, 2^–∆Ct^ [33], after efficiency correction [34] of target and reference gene transcripts (HPRT).

### Immunohistochemistry (IHC)

The expression of IGF2, IGF1R and IGF2R in adrenal samples was evaluated. Paraffin embedded tissue specimens were cut in 5 μm sections, deparaffinized and dehydrated. Antigen-retrieval was performed by microwave treatment in Tris–EDTA Buffer (pH 9.0). The slides were cooled for 1 h at + 4 °C and incubated for 1 h at room temperature (RT) with the primary monoclonal antibodies and incubated overnight at + 4 °C with the primary polyclonal antibodies. The primary monoclonal antibodie to detect IGF1R was purchased from Novus Biologicals (NB110-87052; dilution: 1:500) and the primary polyclonal antibodies to detect IGF2 and IGF2R were purchased from R&D Systems (AF-292-NA; dilution: 1:500) and Santa Cruz Biotech (SC-25462; dilution: 1:50) respectively. The slides were washed and incubated for 30 min at RT with secondary antibodies (Poly-AP-Goat anti-Mouse/Rabbit IgG PowerVision + ; ImmunoVision Technologies) at the concentration provided by the manufacturer. After washing, staining was visualized by a 30 min incubation in new fuchsin solution. Only IGF1R staining was performed and visualized with a Dako Detection System, following a different protocol previously described [25]. All slides were counterstained with hematoxylin and coverslipped. Positive controls included cases of adrenocortical cancer and normal human pancreas with previously proven positivity at IHC for the protein evaluated. Negative controls included omission of the primary antibody and the incubation with secondary antibodies.

The staining was evaluated independently by two investigators and any discrepancy was resolved by a consensus review. The results were interpreted in a semiquantitative manner by using an intensity-proportion scoring system previously described [35]. The score was calculated by the sum of the intensity score and the proportion of the stained cells; this provided a score between 0 and 6. The proportion score was as follows: 0 = no positivity (or less than 10%); + 1 = less than 1/3 tumor cell positivity; + 2 = 1/3 to 2/3 tumor cell positivity; and + 3 = more than 2/3 tumor cell positivity. The intensity score was as follows: + 1 = weak staining; + 2 = intermediate staining; + 3 = strong staining. The score 0 was regarded as negative; 2–3 as low; 4–5 as intermediate and 6 as high. Finally adrenocortical tumors were dichotomously grouped as having intermediate to high expression of the evaluated protein and phospho-proteins (IHC score equal-higher than 4) or not (IHC score lower than 4).

### Drugs and reagents

The dual IGF1R/IR inhibitor linsitinib and the mTOR inhibitors sirolimus and everolimus were purchased from LC Laboratories (Inc. Woburn, MA, USA) and prepared as a 10^−^^3^M stock solution in dimethylsulfoxide (DMSO). Compounds were stored at −20 °C and further diluted in 40% DMSO before the use. Final DMSO concentration, also added as vehicle to controls, was 0.4%.

### Cell lines and culture conditions

The human ACC cell lines H295R and HAC15 were obtained from the American Type Culture Collection (Manassas, VA) and from Dr. W. Rainey (as gift), respectively. Short Tandem Repeat (STR) profiling using a Powerplex Kit (Promega) of H295R gave results consistent with those described in the ATCC database, thus confirming the H295R cell line identity. STR profiling of HAC15 showed that HAC15 has a genetic profile identical to H295R, which is consistent with a previous report by Rainey et al. that HAC15 is a clone of H295R [36]. The cells were cultured as previously described in detail [25] and utilized up to the 15^th^ passage.

### Measurement of total DNA content assay

Measurement of total DNA content per well was used to determine the effects of the compounds on cell proliferation. Cells were plated in 1 ml of medium in 24-well plates at the density required to obtain a 65–70% cell confluence in the control groups at the end of the experiment. The experiments were performed using medium containing high (5% FCS) or low serum (1% FCS). Twenty-four hours later compounds were added to wells in quadruplicate, medium was refreshed at day 3 and fresh compounds were added again. After 3 or 6 days of treatment with the selected compounds, cells were harvested for DNA measurement, as a measure of cell number. All controls were vehicle treated. Measurement of total DNA content was previously described in detail [37].

### Apoptosis assay

Apoptosis has been studied using two methods: “DNA fragmentation assay” and “Muse^TM^ Annexin V & Dead Cell Kit”.

#### DNA fragmentation assay

The cells were plated in 24-well plates and treated as above described for the cell proliferation assay. After 24 h compounds or vehicle were added and after 3 days of incubation, DNA fragmentation was determined using a commercially available ELISA kit (Roche Diagnostic GmbH, Penzberg, Germany). The standard protocol supplied by the manufacturer was used. The same plates were also analyzed for the measurement of total DNA content. The amount of DNA-fragmentation (apoptosis) was corrected for the total DNA content in each well.

#### Muse^TM^ Annexin V & Dead Cell Kit (Millipore, Germany)

Cells were plated in 12-well plates at the density necessary to obtain a 65–70% cell confluence in the control groups at the end of the experiment. Twenty-four hours later, sirolimus was added to wells in duplicate. Control groups were vehicle-treated. After seventy-two hours of treatment, cells were harvested by gentle trypsinization and processed for staining according to the protocol provided by the supplier of the assay. The experiments were repeated twice.

### Cell cycle assay

The effects of compounds on cell cycle progression were evaluated using the “Muse^TM^ Cell Cycle Assay” (Millipore, Germany). Cells were plated in 12-well plates at the density necessary to obtain a 65–70% cell confluence in the control groups at the end of the experiment. Twenty-four hours later sirolimus was added to wells in duplicate. Control groups were vehicle-treated. After seventy-two hours of treatment, cells were harvested by gentle trypsinization and processed for fixation and staining according to the protocol provided by the supplier of the assay. The experiments were repeated twice.

### Statistical analysis

All the experiments were carried out at least three times, with the exception of the apoptosis assays and cell cycle assay that were performed twice. The repeated experiments gave comparable results. For the statistical analysis, statistical software SPSS (SPSS 15.0; SPSS Inc., Chicago, IL) and GraphPad Prism 5.0 (GraphPhad Software, San Diego, CA) were used. The Spearman’s rank coefficient (rho) was used to test correlation.

We used non-parametric tests to evaluate the differences among groups (Mann–Whitney and Kruskall–Wallis). The comparative statistical evaluations among treatment groups were performed by ANOVA, followed by a multiple comparison test (Newman–Keuls).

## Results

### Study population

This study included samples from seventeen patients with ACC (main clinical characteristics reported in Table 1). Only two of the included ACC patients were children in which the presence of a genetic cause was not known (case 6 and 8; 9.5 and 4.2 years old respectively).

**Table 1:** IGF2, IGF1R and IGF2R protein expression in 17 adrenocortical cancer samples

Patient number	Sex	Weiss	Hormonal secretion	IGF2 protein expression		IGF1R protein expression		IGF2R protein expression	
				Score	Considerable expression	Score	Considerable expression	Score	Considerable expression
**1**	F	3	C	4	Yes	5	Yes	4	Yes
**2**	F	5	C and A	6	Yes	4	Yes	5	Yes
**3**	F	9	A	6	Yes	3	No	4	Yes
**4**	M	6	C and A	3	No	6	Yes	5	Yes
**5**	F	6	A	5	Yes	5	Yes	5	Yes
**6**	F	7	C and A	6	Yes	6	Yes	5	Yes
**7**	M	8	none	4	Yes	5	Yes	6	Yes
**8**	F	4	A	6	Yes	3	No	5	Yes
**9**	F	7	none	4	Yes	3	No	5	Yes
**10**	F	3	none	3	No	4	yes	5	Yes
**11**	F	7	C and A	6	Yes	4	Yes	5	Yes
**12**	F	5	A	5	Yes	6	Yes	6	Yes
**13**	F	7	none	4	Yes	3	No	4	Yes
**14**	F	8	none	6	Yes	3	No	5	Yes
**15**	M	4	A	6	Yes	4	Yes	6	Yes
**16**	F	6	none	6	Yes	2	No	5	Yes
**17**	M	6	C	3	No	5	Yes	6	Yes
		Median 6; Range 3–9		Median 5; Range 3–6	Frequency 14/17 (82%)	Median 4; Range 2–6	Frequency 11/17 (65%)	Median 5; Range 4–5	Frequency 17/17 (100%)

*F* female, *M* male, *C* cortisol, *A* androgens

To describe the IGF pathway, the protein expression levels of IGF2, IGF1R and IGF2R were evaluated by IHC in the ACC samples. In twelve of these samples, the mRNA expression levels of *IGF1*, *IGF2*, *IGF1R*, *IRA*, *IRB*, *IGF2R*, *IGFBP1, 2, 3* and *6* were evaluated by RT-qPCR.

### mRNA expression of the components of the IGF pathway in human ACC and NA samples

**Fig. 1:**
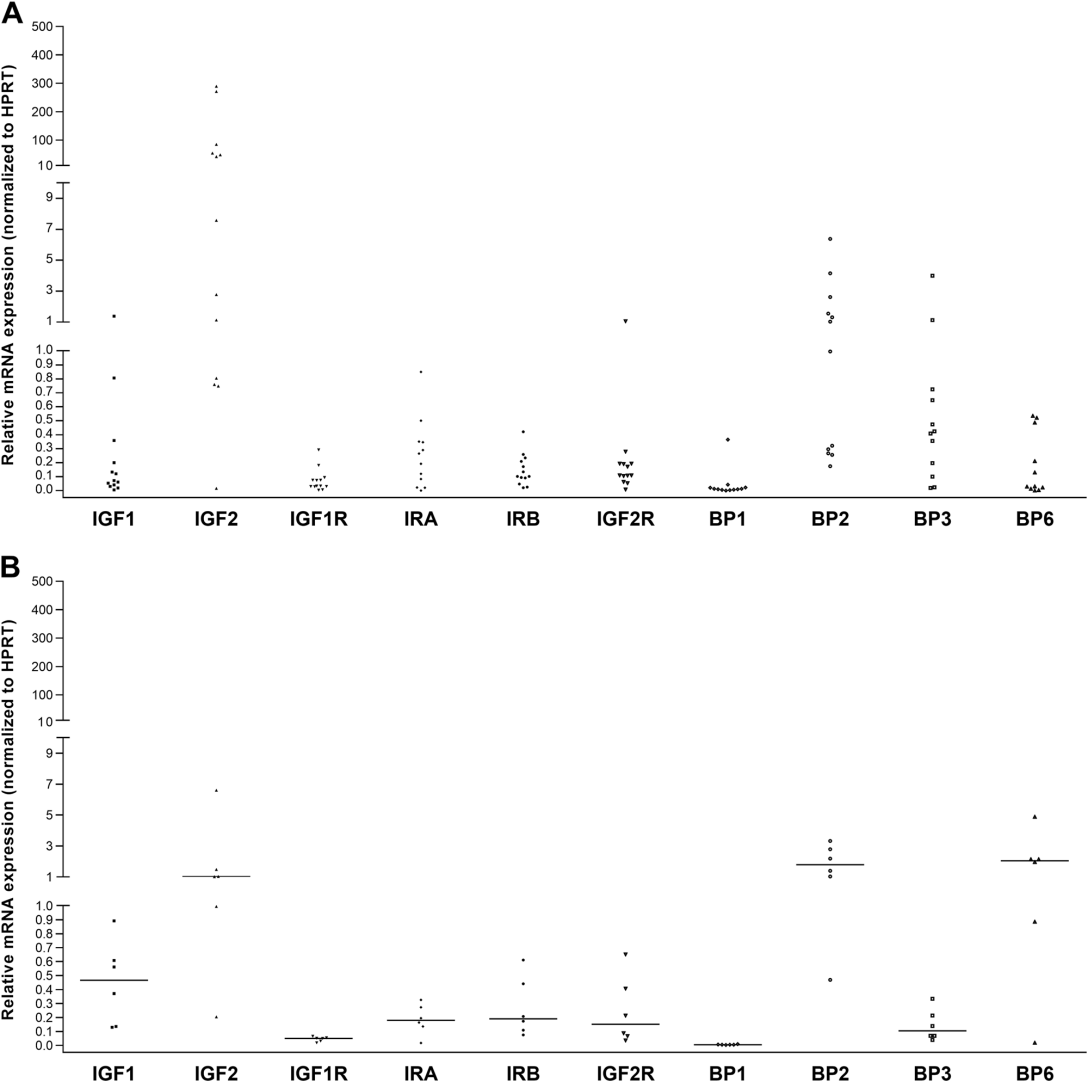
mRNA expression levels of the main components of the IGFs and mTOR pathways (expressed as relative mRNA expression as normalized to the housekeeping gene HPRT) in a series of 17 human ACC samples **a** and in a series of 6 normal adrenals **b**

The mRNA expression of several components of the IGF pathway was evaluated by RT-qPCR in 12 ACC samples and in 6 NA samples. As shown in Fig. 1, the expression levels of most of these IGF pathway components are quite variable in the different samples evaluated, although a high relative expression of *IGF2* was found in the majority of samples observed (mean 66,8 ± 106,4; median levels 24.82; range 0.01–289.68). As compared with other receptors evaluated, the receptor expressed at highest levels within tumors was *IRA* in 7 of 12 samples (58.3%), *IGF2R* in 3 (25%), *IGF1R* in one (8.3%) and *IRB* in the remaining one (8.3%). Considering only the tyrosine-kinase receptors, *IRA* was the receptor expressed at highest levels in the majority of samples (83%). Mean levels of *IRA* were significantly higher than mean levels of *IGF1R* (0.25 ± 0.26 vs. 0.07 ± 0.09; *p* < 0.05). In all the evaluated samples, excepted for three cases, *IRA/IRB* ratio was higher than 1 (2.19 ± 1.59). As compared with other IGFBPs evaluated, the *IGFBP* expressed at highest levels within tumors was *IGFBP2* in 7 of 12 samples (58.3%); *IGFBP3* in 4 (33.3%) and *IGFBP6* in only one case (8.3%). Mean levels of *IGFBP2* were significantly higher than the mean level of *IGFBP1* and *IGFBP6* (1.16 ± 1.9 *versus* 0.04 ± 0.1; *p* < 0.01 and vs. 0.18 ± 0.07; *p* < 0.05, respectively). A negative correlation was found between *IGF2* and *IGFBP6* (rho: −0.8; *p* < 0.003), whereas a positive correlation was found between *IGF1R* and *IGF2R* (rho: 0.7; *p* < 0.009); I*GF1R* and *IRB* (rho: 0.8; *p* < 0.003); *IGF1R* and *IGFBP1* (rho: 0.8; *p* < 0.001) and *IRA* and *IGFBP2* (rho: 0.8; *p* < 0.003). No relationship was observed between the mRNA levels of the IGF components and any clinical parameter evaluated including hormone production, Weiss score, mitotic index and TNM.

Mean levels of *IGF1* and *IGFBP6* were significantly lower in ACCs as compared with NAs (0.3 ± 0.4 versus 0.5 ± 0.3; *p* < 0.05 and 0.2 ± 0.2 vs. 2.0 ± 1.6, *p* < 0.01, respectively). Mean levels of *IGF2* were considerably higher in ACCs as compared with NAs (66.8 ± 103.6 vs. 1.9 ± 2.3; *p* < 0.05), but this difference did not reach statistical significance, probably as a consequence of the small sample size and the high variation of *IGF2* levels within the tumor samples. Comparing the expression of the evaluated components of the IGF pathway in the 12 ACCs evaluated to the median value of each component in the NA, we observed an over expression of: *IRA* in 7 cases (58%); *IRB* in 4 (33,3%); *IGF1* in 2 (17%); *IGF2* in 9 (75%); *IGF1R* in 5 (41.6%); *IGF2R* in 5 (41.6%); *IGFBP1* in 10 (83.3%); *IGFBP2* in 3 (25%); *IGFBP3* in 9 (75%) and *IGFBP6* in none. In addition, 9 ACC samples showed an *IRA*-*IRB* ratio higher than the median value observed in normal adrenals.

### Protein expression of the components of the IGF pathway in human ACC samples

**Fig. 2:**
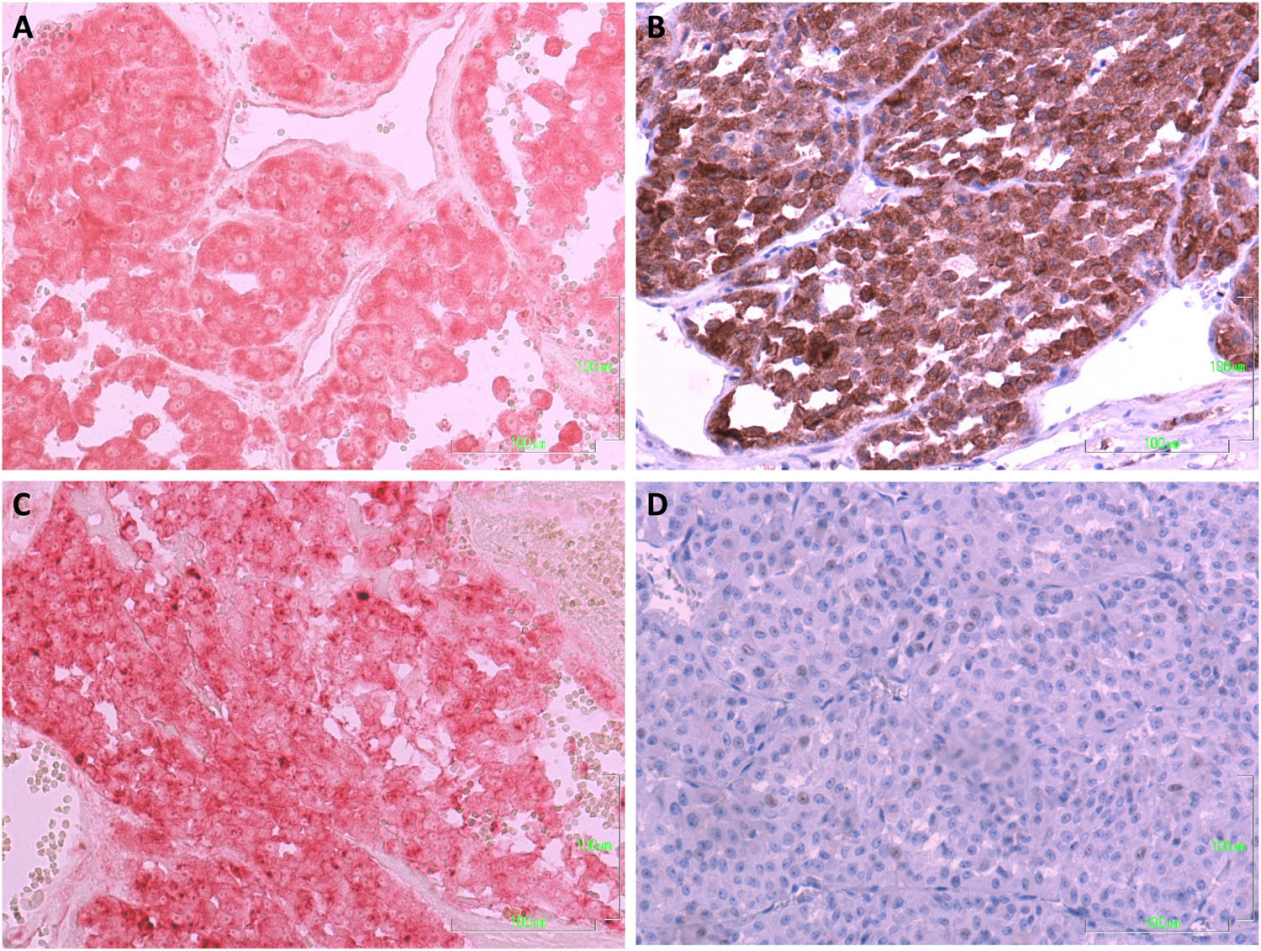
Immunohistochemical detection of IGF2 **a**, IGF1R **b** and IGF2R **c** in a case of human adrenocortical carcinoma. **d** shows the absence of staining in the negative control. Magnification, ×200

The protein expression of IGF2, IGF1R and IGF2R was evaluated by IHC in 17 human ACCs. Table 1 summarizes the results of the IHC and the main clinical features of the evaluated patients. An intermediate to high staining for IGF2 (82%; median score 5; range 3–6) and IGF1R (65%; median score 4; range 2–6) was observed in most tumor tissues and for IGF2R (median score 5; range 4–6) in all ACCs. No correlations were observed between the expression of the different proteins that were evaluated and between these proteins and the main clinical-pathological characteristics of the corresponding patients. No correlations were observed between the protein and mRNA expression of IGF2, IGF1R and IGF2R although a trend to positive correlation was found between IGF2 protein and mRNA expression. Figure 2 shows an exemplary case of immunostaining in ACC. No particular expression has been observed in the two childhood ACC included in this series.

### Effects of dual IGF1R/IR inhibitor in human adrenocortical cell lines

**Fig. 3:**
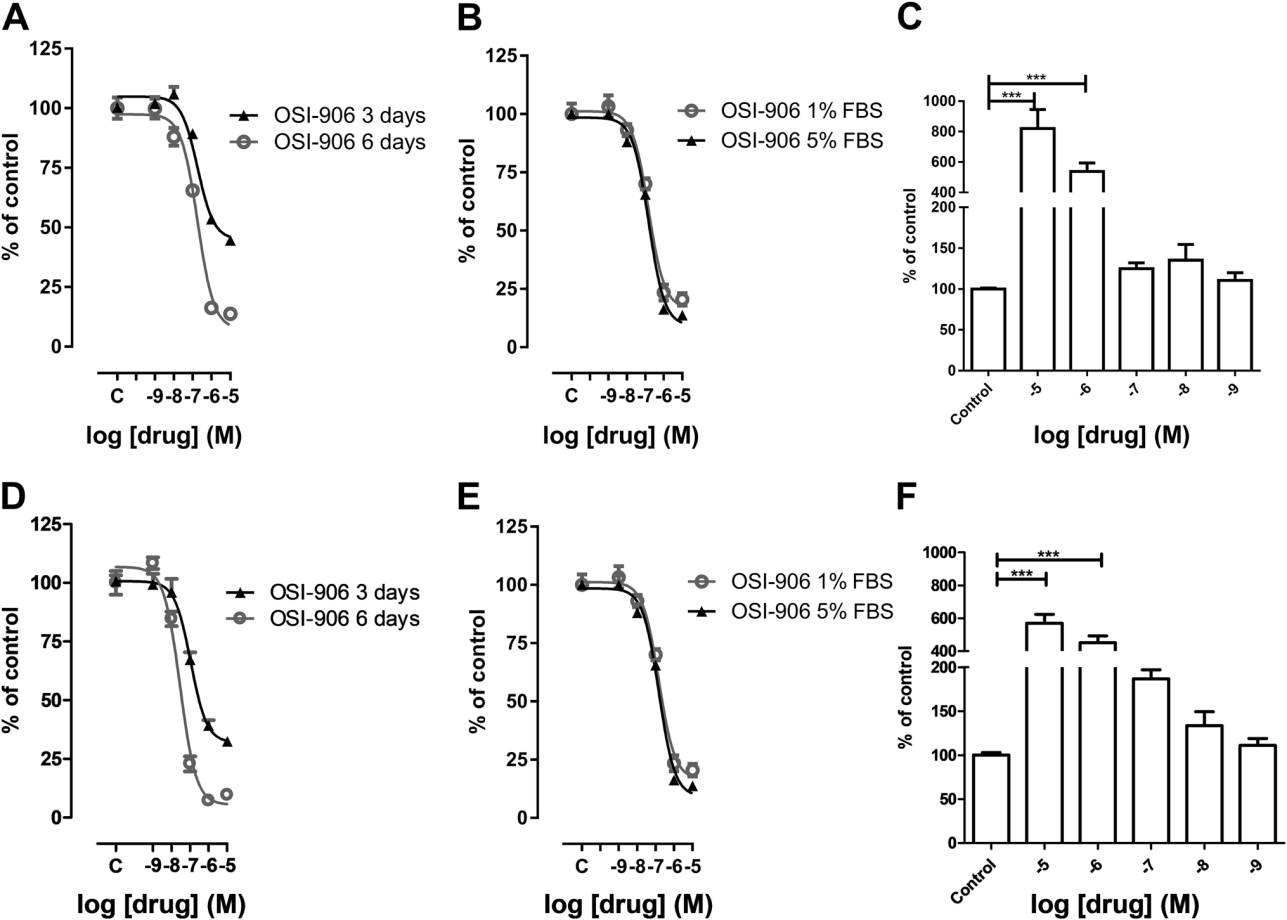
Dose and time-dependent effect of linsitinib (OSI-906) treatment on H295R **a**, **b** and HAC15 **d**, **e** cell proliferation, expressed as DNA content/well after 3 days and 6 days **a**, **d** and after 6 days in medium with high or low serum **b**, **e**. Dose-dependent effects of 3-day treatment with linsitinib on apoptosis of H295R **c** and HAC15 **f** cells, expressed as DNA fragmentation (normalized to the DNA content of each well). Data are expressed as the percentage of control and represent the mean ± SEM. Control is set as 100%. ***p < 0.001

In both H295R and HAC15 cell lines linsitinib inhibited cell proliferation in a dose- and time-dependent manner (Fig. 3a, d). Linsitinib was slightly, but significantly, more potent in inhibiting cell proliferation in HAC15 compared to H295R. After 6 days of treatment in full medium the IC_50_ of linsitinib in H295R was 1.5 × 10^−^^7^ M and in HAC15 2.9 × 10^−8^ M (*p* < 0.01). The maximal inhibition observed in H295R and HAC15 was 90 and 95%, respectively (not statistically significant; *p* = 0.3). In both the H295R and HAC15 cells the potency of linsitinib and the maximal inhibition observed were similar in cell cultured in medium with high serum compared with cells cultured in medium with low serum (Fig. 3b, e). At the condition tested, linsitinib induced DNA-fragmentation in a dose-dependent manner in both H295R and HAC15 (Fig. 3c, f).

### Effects of the dual IGF1R/IR inhibitor linsitinib in combination with mTOR inhibitors on human ACC cells

**Fig. 4:**
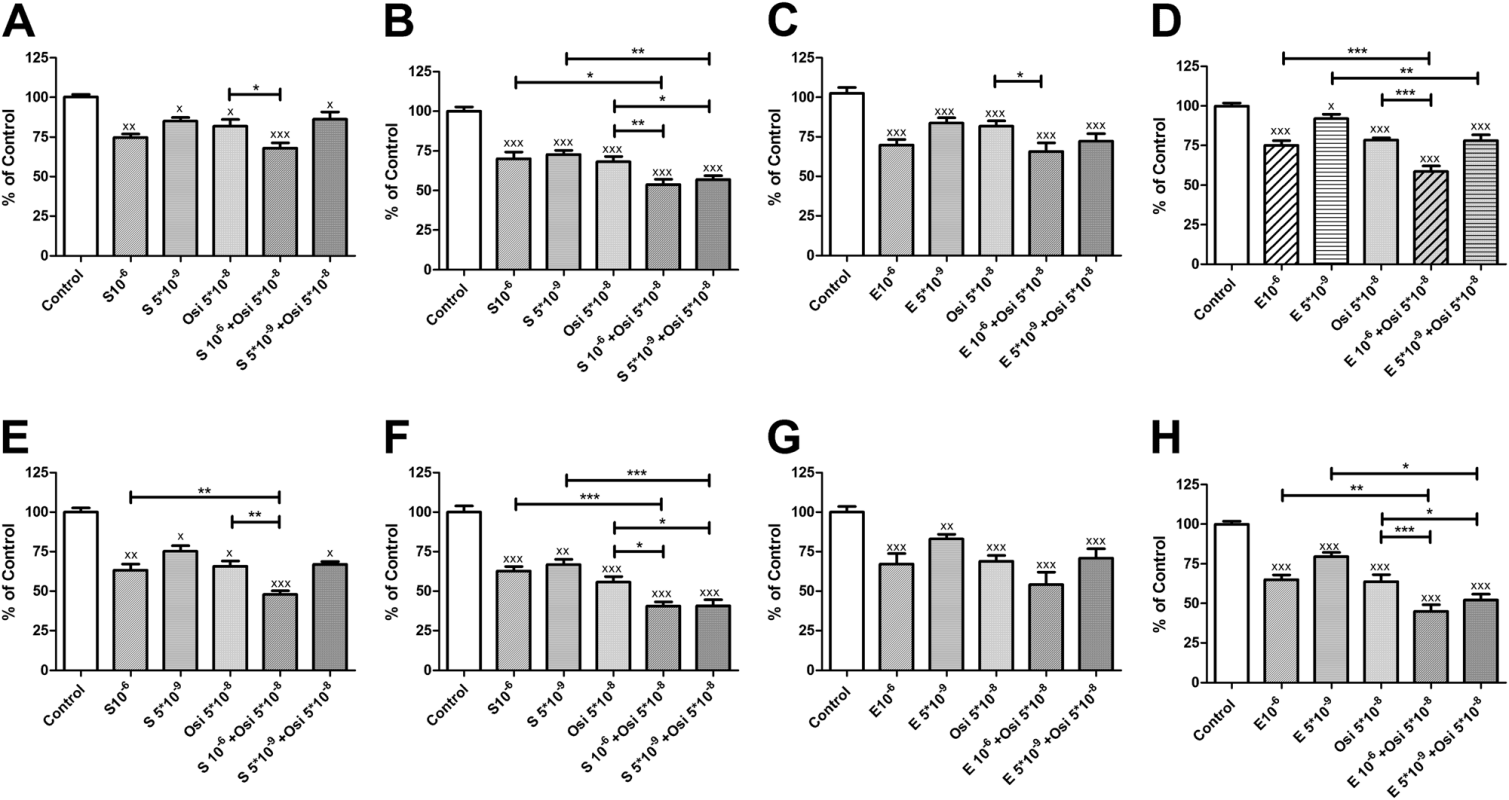
Effect of linsitinib (OSI-906 indicated as Osi), alone or in combination with the mTOR inhibitors sirolimus (S) or everolimus (E), on H295R **a**–**d** and HAC15 **e**–**h** cell proliferation. Results are expressed as DNA content/well. Two different conditions have been tested: medium with high **a**, **c**, **e**, **g** or low serum **b**, **d**, **f**, **h**. The data are expressed as the percentage of control and represent the mean ± SEM. Control is set as 100%. *p < 0.05; **p < 0.01; ***p < 0.001; ^x^*p* < 0.05 vs. control; ^xx^*p* < 0.01 vs. control; ^xxx^*p* < 0.001 vs. control

Sirolumus and everolimus inhibited cell proliferation in H295R and HAC15 cells in a dose-dependent manner in both experimental conditions tested (high *versus* low serum concentration medium) data not shown. Sirolimus was slightly, but not significantly, more potent than everolimus. The potency of both compounds was similar in medium containing either high or low serum concentration. Selected doses of sirolimus or everolimus combined with linsitinib 5 × 10^−8^ M had statistically significant additive effect on cell proliferation (Fig. 4). Particularly both concentrations used of sirolimus and everolimus showed additive effect with linsitinib in inhibiting H295R and HAC cell proliferation when tested in medium containing low serum concentration (Fig. 4b, d, f, h). Only the highest concentrations used of mTOR inhibitors (10^−^^6^ M) showed some additive effect with linsitinib in inhibiting H295R and HAC cell proliferation when tested in medium containing high serum concentration (Fig. 4a, c, e, g).

**Fig. 5:**
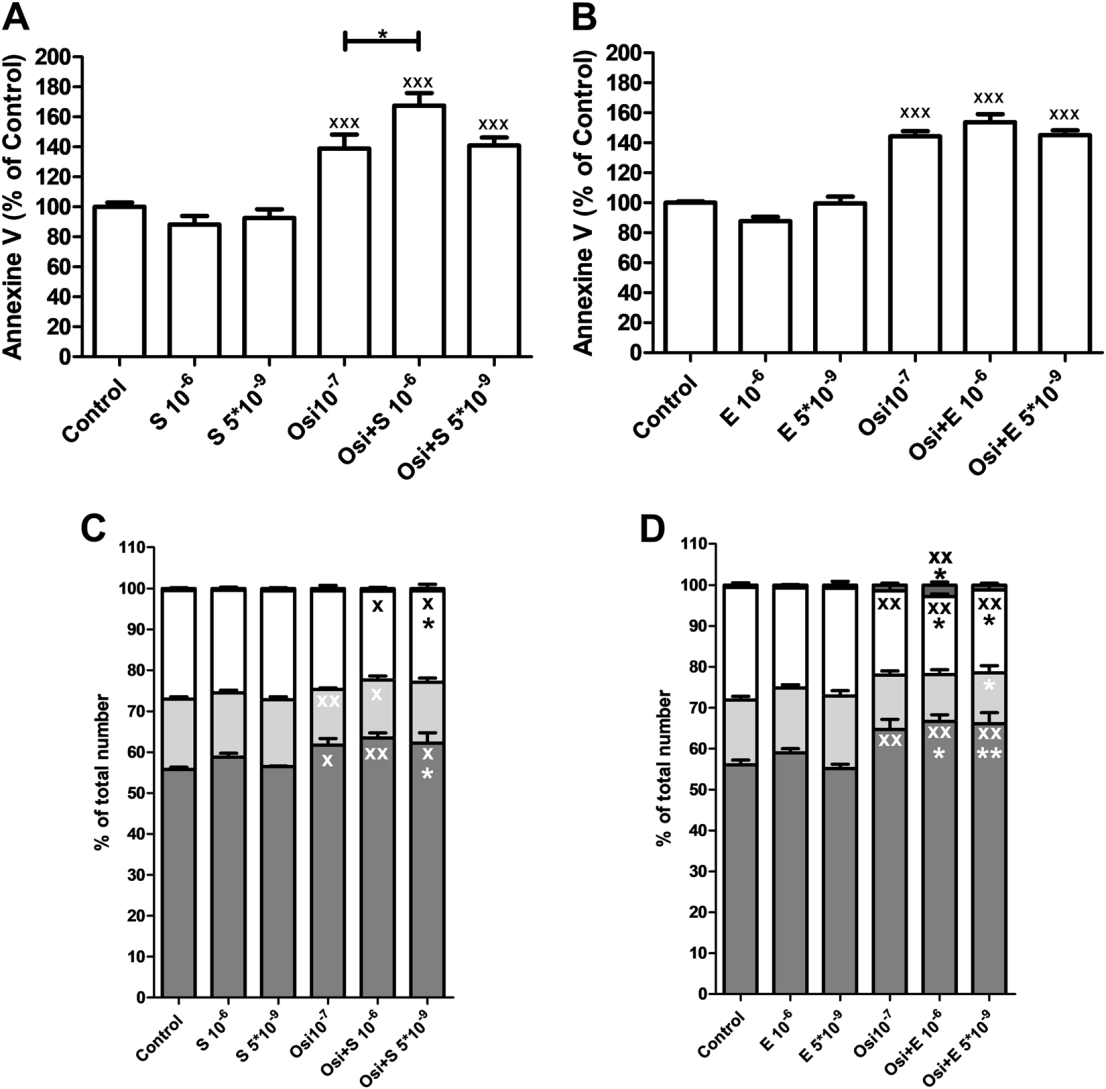
**a**, **b**: Effect of linsitinib (OSI-906 indicated as Osi), alone or in combination with the mTOR inhibitors sirolimus [(S); **a**] or everolimus [(E); **b**], on Annexin V as a measure of induction of apoptosis in H295R cell line. The data are expressed as the percentage of control and represent the mean ± SEM. Control is set as 100%. *p < 0.05 vs. Osi alone; ^xxx^*p* < 0.001 vs. control. **c**, **d** Effect of linsitinib (Osi), alone or in combination with the mTOR inhibitors sirolimus **c** or everolimus **d**, on cell cycle in H295R cell line. The data are expressed as mean ± SEM. *p < 0.05 and **p < 0.01 vs. S **c** or E **d** alone; ^x^*p* < 0.05 and ^xx^*p* < 0.01 vs. control

At the condition tested, only the highest concentrations used of sirolimus (10^−6^ M) showed significant additive effect with linsitinib in increasing annexin V, used as measure of apoptosis, in H295R (Fig. 5a). Everolimus did not show a statistically significant additive effect in increasing annexin V in H295R (Fig. 5b).

Linsitinib (10^−^^7^ M) alone or in combination with sirolimus 10^−^^6^M or 5 × 10^−9^ M significantly increased the proportion of cells in G_0_/G_1_ phase of the cell cycle (*p* < 0.05, *p* < 0.05 and *p* < 0.01 respectively). Linsitinib in combination with sirolimus 10^−^^6^ M or 5 × 10^−^^9^ M significantly reduced the proportion of G_2_/M (*p* < 0.05) (Fig. 5c). Additionally the combined treatment with linsitinib and sirolimus showed a trend to have additive effects in inducing G1-cell cycle block. Statistically significant additive effects in increasing the proportion of cells in G_0_/G_1_ phase (*p* < 0.05) and reducing the proportion of cells in G_2_/M phase (*p* < 0.05) were observed when combining linsitinib 10^−^^7^ M and sirolimus 5 × 10^−^^9^ M as compared with sirolimus alone (Fig. 5c).

Linsitinib (10^−^^7^ M) alone or in combination with everolimus 10^−^^6^M or 5 × 10^−^^9^ M significantly increased the proportion of cells in G_0_/G_1_ phase of the cell cycle (*p* < 0.01). Linsitinib alone or in combination with everolimus 10^−^^6^ M or 5 × 10^−^^9^ M significantly reduced the proportion of cells in G_2_/M phase (*p* < 0.01) (Fig. 5d). Additionally, the combined treatment with linsitinib and everolimus showed significant additive effects in inducing G1-cell cycle block. Particularly, when combining linsitinib 10^−^^7^ M and everolimus 10^−^^6^ M or 5 × 10^−^^9^ M, statistically significant additive effects in increasing the proportion of cells in G_0_/G_1_ phase (*p* < 0.05 and *p* < 0.01, respectively) and in reducing the proportion of G_2_/M (*p* < 0.05) as compared with everolimus alone were observed (Fig. 5d).

## Discussion

The results of this study show that the majority of ACC express IGF2, IGF1R and IGF2R mRNA and protein and demonstrate IRA mRNA expression in these tumors, suggesting that factors such as IGF2R and IRA, not well described before, could interact with IGF2, potentially modulating the role of IGF2 in adrenocortical tumorigenesis. Mean levels of IGF1 and IGFBP6 were significantly lower in ACCs as compared with NAs. Additionally, this study demonstrates that treatment of human ACC cells with OSI-906, a dual IGF1R/IR inhibitor, reduces cell proliferation and that combined treatment with linsitinib and mTOR inhibitors can have additive antiproliferative effects.

A high mRNA and protein expression of IGF2 is found in most evaluated samples, in agreement with the already well-known IGF2 overexpression in 70-90% of ACCs [8–17]. IGF1R protein expression was demonstrated in all the evaluated ACC samples and an intermediate-to-high staining was observed in more than 50% of cases. These data are in agreement with previous studies describing IGF1R expression in most ACCs [11, 14, 38]. The protein expression of IR in most ACCs has been previously described as well [38], however, to the best of our knowledge, the differential expression of IRA and IRB isoforms of the IR in ACCs, has never been explored. Unfortunately, to the best of our knowledge, there are currently no antibodies available to distinguish between the IR isoforms. We could therefore only evaluate IR isoform expression at mRNA level. While IRB is considered as the main mediator of metabolic effects of insulin and IGFs in adult tissue, IRA is an isoform of the IR, predominantly expressed during fetal development and is considered as an important mediator of growth-promoting effects of insulin and IGFs [19]. IRA has a higher affinity for IGF2 compared with the IGF1R and its expression in malignant tumor tissue has been suggested to be involved in cancer development [19]. To our knowledge this is the first study demonstrating the presence of IRA in ACCs and showing that in these cancers, IRA is often expressed at higher level compared with IGF1R and IRB. The expression of IGF2 and the IGF1R has suggested a potential role of an IGF2-IGF1R autocrine loop in adrenocortical tumorigenesis [39]. The current study suggests a role of IRA as potential additional mediator of the IGF2 effects in ACC. However, in addition to the tyrosine kinase receptors involved in the IGF pathway, also IGF2R and IGFBPs could play a role in modulating the IGFs effects. The IGF2R serves a function in the degradation of IGF2, intracellular trafficking of lysosomal enzymes and activation of transforming growth factor beta. Down-regulation of IGF2R has been found in some type of cancers and it has been suggested that IGF2R could play a role as a tumor suppressor gene in some malignancies [40, 41]. Loss of heterozygosis at the locus of IGF2R gene has been reported to be a frequent event in ACC, supporting a potential role of IGF2R as a tumor suppressor gene also in ACC development [42]. However, a low protein expression of IGF2R in ACC has never been described. Conversely, the current study demonstrates the presence of a high IGF2R protein expression in most ACCs, suggesting that a high level of IGF2R protein might counteract the growth-stimulating effects of IGF2 in adrenocortical tumorigenesis. In line with previously published data, in the current study a variable expression of IGFBPs was found in ACCs [10, 43]. Several correlations between the mRNA expression of different components of the IGF pathway were found suggesting the existence of common mechanisms of regulation. However no correlations with clinical-pathological parameters were found. This lack of correlation might be related to the small sample size and to the complexity of the IGF pathway. Among the IGFBPs evaluated, the IGFBP expressed at higher levels was IGFBP2, whereas IGFBP6 was expressed at lowest level. Additionally the expression of IGFBP6 was significantly lower in ACCs than in NAs. Therefore, whether high IGFBP2 and/or low IGFBP6 could play a role in the regulation of IGF pathway in adrenocortical tumorigenesis deserves further investigation. Additionally the IGFBP expressed at highest levels within ACC was IGFBP2, which has been recently suggested as a potential target for treatment in some type of cancers [44]. Therefore, in future studies it might be interesting to better explore the role of this and other IGFBPs as potential target for treatment in ACC as well.

The IGF pathway has been considered as one of the most promising targets for a novel medical treatment modality in patients with ACC [11, 14, 26, 45]. In preclinical models of ACC, two types of drugs targeting the IGF1R, i.e., NVP-AEW541, a selective IGF1R kinase inhibitor and IMC-12, an IGF1R antibody, have been reported to have antiproliferative effects [11, 14], thus encouraging the development of clinical trials in ACC patients using drugs targeting the IGF pathway. The current study confirms that linsitinib (OSI-906), an IGF1R/IR inhibitor, inhibits the proliferation of the human ACC cell lines H295R and HAC15 in vitro already at a concentration lower than the concentrations reached in vivo in humans (about 5 × 10^−^^6^ M). However linsitinib has been recently tested in ACC patients in a phase III clinical trial (NCT00924989). The results of this study have been recently published [46] showing that only a very small subgroup of patients seems to benefit from treatment with this drug, and improvements in overall or progression-free survival were not observed. These apparent controversial results between preclinical and clinical studies, could be explained by several reasons. First, it might indicate that our preclinical models are not enough representative for the population of patients with ACC, because these tumors are heterogeneous. The role of the IGF pathway as a potential target for treatment in ACC might have been overestimated, as suggested by the fact that up-to-date in vivo experiments demonstrated that isolated IGF2 overexpression has no oncogenic potential [47]. Since disappointing results emerged in clinical trials adopting different types of drugs targeting the IGF pathway in different types of malignancies, despite apparently promising preclinical data [48], it could be hypothesized that current strategies adopted to target this pathway might still be inadequate. Indeed, biomarkers that can predict tumor response to IGF-targeting drugs, that might drive the selection of patient candidates to these drugs, have not been identified yet. Additionally, the complexity of the system could have been underestimated (such as the expression of potential regulators of the IGF pathway, as IGF2R in ACC) and the existence of interfering factors may not have been characterized yet. For example, in case of ACC patients the potential pharmacokinetic interactions between mitotane and drugs acting on the IGF pathway should be better investigated. Mitotane is a strong inducer of CYP3A4 and was shown to decrease bioavailability of sunitinib in patients with ACC [49]. Finally, targeting only the IGF pathway might not be sufficient to suppress cell growth because other pathways, that in part also interact with the IGF pathway (e.g. the mTOR pathway) are still activated. As such, before to finally declare a “game over” [47] for the role of IGF2 in adrenocortical tumorigenesis and as potential target for novel treatment in ACC patients, it could be probably useful to return to the bench and try to better explore the IGF pathway in its whole complexity. In line with this, the results of the current study point out that ACC express components of the IGF pathway, such as IRA and IGF2R, that have not been considered before.

In a previous study from our group, it was demonstrated that mTOR inhibitors inhibit cell proliferation in H295R and SW13 human ACC cell lines, but in H295R, probably as consequence of the IGF2 overexpression, this treatment could activate two potential pathways of escape to treatment with traditional mTOR inhibitors, i.e. the AKT and ERK pathways [25]. These data provide the rational for experiments combining mTOR inhibitors and drugs targeting the IGF pathway in ACC. In the current study the effects of linsitinib in combination with mTOR inhibitors were evaluated and the results of these experiments demonstrated that these compounds can have additive antiproliferative effects in some of the tested conditions. Particularly, additive antiproliferative effects were more pronounced when the experiments were performed using medium with low serum, suggesting that cell environment and the presence of growth factors different from IGF2 could influence the effects of these combination of compounds. These results are in line with a recently published phase I study demonstrating that a subgroup (about 40%) of ACC patients treated with cixutumumab (IGF1R inhibitor) and temsirolimus experienced a long term disease stabilization [28]. These results suggest that treatment strategies combining mTOR inhibitors and linsitinib warrant further investigation, although considering the heterogeneous expression of the main components of the IGF pathway in the different ACC samples, the apparently modest antiproliferative effects observed at a low concentration of these compounds as well as the potential limits of the used human cell lines as model of human ACC, caution is recommended before to move from the bench to the bedside.

A potential limitation of the current study is the small sample size. Although the number of samples included is reasonable considering the rarity of ACC, the reported results require confirmation in larger series of samples.

In conclusion, the present study describes the IGF pathway in ACC and explores the response to the combined treatment with the dual IGF1-/IR inhibitor linsitinib, and mTOR inhibitors in in-vitro models of ACC, demonstrating that human ACC express IGF2R and IRA which, in addition to IGF2 and its receptor IGF1R, might modulate the IGFs effects and linsitinib and mTOR inhibitors have additive antiproliferative effects.

## Supplementary information

Supplementary table.
