# Supplementary material for: IGF and mTOR pathway expression and in vitro effects of linsitinib and mTOR inhibitors in adrenocortical cancer
Source: Endocrine. 2019 Mar 5;64(3):673–84. doi: 10.1007/s12020-019-01869-1 (PMC6551351; doi:10.1007/s12020-019-01869-1)
Supplement: Supplementary file 1 — Supplementary table. [file 12020_2019_1869_MOESM1_ESM.doc]

Supplemental material

Table 1
